# Zika virus infection in human placental tissue explants is enhanced in the presence of dengue virus antibodies in-vitro

**DOI:** 10.1038/s41426-018-0199-6

**Published:** 2018-12-01

**Authors:** Kyra Hermanns, Claudia Göhner, Anne Kopp, Andre Schmidt, Waltraut M. Merz, Udo R. Markert, Sandra Junglen, Christian Drosten

**Affiliations:** 1 Institute of Virology, Campus Charité Mitte, Charité – Universitätsmedizin Berlin, Berlin, Germany; 2 Institute of Virology, University of Bonn Medical Centre, Bonn, Germany; 3 Placenta Laboratory, Department of Obstetrics, University Hospital Jena, Jena, Germany; 4 Department of Obstetrics and Prenatal Medicine, University Bonn Medical School, Bonn, Germany; 5 Berlin Institute of Health, Berlin, Germany

## Abstract

The current Zika virus (ZIKV) outbreak is associated with neurological malformations and disorders in neonates. Areas of increased incidence of malformations may overlap with dengue-hyperendemic areas. ZIKV infection is enhanced by antibodies against dengue virus (DENV) in cell culture and inbred mice. Sufficiently powered clinical studies or primate studies addressing the enhancement of fetal ZIKV infection after previous dengue infection are not available. The human placenta is susceptible to ZIKV in vitro, but it is unknown whether antibody-dependent enhancement of ZIKV infection occurs at the placental barrier. Here we studied ZIKV infection in placental tissue in the presence of DENV-immune sera. Explants from the amniochorionic membrane, the chorionic villi, and the maternal decidua were infected with ZIKV in the presence of DENV type 1-, 2-, or 4-immune sera, or controls. Presence of DENV antibodies of any type enhanced the percentage of successful infections of organ explants between 1.42- and 2.67-fold, and led to a faster replication as well as significantly increased virus production. No enhancement was seen with yellow fever or chikungunya virus control sera. Pre-existing DENV antibodies may pose an increased risk of trans-placental ZIKV transmission.

## Introduction

Zika virus (ZIKV) belongs to the genus *Flavivirus* (family: *Flaviviridae*) and is related to other important human pathogens such as dengue virus (DENV), yellow fever virus (YFV) and West Nile virus. The associated disease is characterized by flu-like symptoms, conjunctivitis and rash, and is often mild or asymptomatic.

A distinct ZIKV lineage was first detected in an outbreak in Yap island in 2007^1^, and subsequently affected the population of French Polynesia in 2013^2^. The lineage adapted to urban circulation in South East Asia before emergence in the Pacific region and is thought to have been introduced to Brazil around 2013 to 2014, from where it spread within the Americas^3,4^. In Brazil, the resulting ZIKV epidemic in a non-immune population correlated with a rise in adverse pregnancy outcomes, notably an increase in microcephaly and other congenital conditions, first noticed in 2015^5^. Retrospectively, evidence for maternal–fetal transmission with cerebral malformations was also obtained for the outbreak in French Polynesia^6^.

French Polynesia and Brazil have high prevalence rates of DENV infection. In French Polynesia, over 80% of the population have pre-existing immunity against at least one DENV serotype^7^. In Brazil, reported seroprevalence rates in cities range from 30% in Goiânia (Centre-West) to 98% in Mossoró (Northeast)^8^. Most microcephaly cases after the emergence of ZIKV were reported in the Northeast of Brazil^9^.

DENV infections are caused by four distinct viral serotypes that do not induce mutual cross-protective immunity. Antibodies from a previous DENV infection can lead to antibody-dependent enhancement (ADE) during secondary infection caused by a heterologous serotype. Cross-reactive, non-neutralizing antibodies bind to the virus and induce viral uptake into Fcγ receptor-bearing target cells^10^.

The E protein of ZIKV is related to the E protein of DENV, to the extent that antibodies targeting the E-dimer epitope from some patients with previous DENV infections can cross-neutralize ZIKV. This cross-neutralization is seen in tests based on Vero cells that do not express Fcγ receptors^11,12^. When using Fcγ receptor-bearing cells, in vitro ZIKV infection undergoes ADE in the presence of anti-DENV sera^13^. Increased ZIKV-associated morbidity and mortality was seen when mice were infected with ZIKV in presence of infused DENV antibodies^13^. However, in rhesus macaques no differences in pathogenicity were seen in adult animals that were immune or naïve against DENV or YFV^14,15^. None of these studies examined the influence of pre-existing DENV immunity on maternal–fetal transmission or placental infection.

The human placenta defines the materno-fetal interface that blood-borne viruses must overcome before infecting the fetus. The placental chorionic villi are separated from the maternal blood by a syncytiotrophoblast layer that expresses neonatal immunoglobulin Fc receptors (FcRn), as well as Fcγ receptors^16,17^. During pregnancy, immunoglobulin G (IgG) is transported from the maternal blood to the fetal circulation. The FcRn facilitates receptor-mediated transcytosis of IgG through the syncytiotrophoblast layer^17,18^. Although the syncytiotrophoblast layer seems resistant to ZIKV infection, cytotrophoblast and immune cells (Hofbauer cells, decidual macrophages) of the villous and basal decidual compartment, as well as decidual cells and amnion epithelial cells that line the amniotic sac have been found to be susceptible to ZIKV infection^19,20^. DENV antibodies could enhance ZIKV infection in these placental and paraplacental compartments, which would increase the chances of infection of fetal tissues. However, ADE has not been studied in human placental tissues. Here we provide observational evidence that DENV-immune serum confers ADE of ZIKV infection in fresh tissue explants from three relevant compartments of term human placentae.

## Results

### Cell culture growth kinetics

**Fig. 1:**
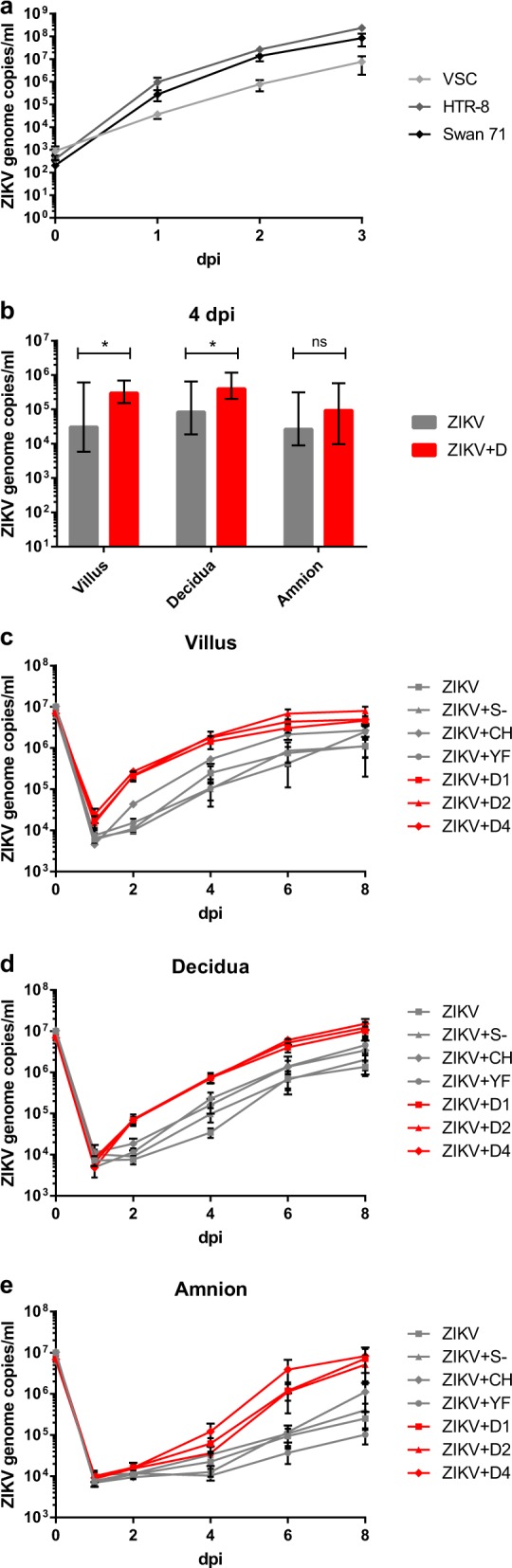
Replication and DENV-specific ADE in cell cultures and placental explants. **a** ZIKV replication in placental cell lines. Cells were infected with ZIKV at MOI of 0.1. ZIKV genome RNA concentrations were measured by real-time RT-PCR for 3 days after infection. Each datum point represents the mean of triplicates with SD. **b** ZIKV replication kinetics in different placental tissue explants and ADE by DENV antibodies. Placental villus, maternal decidua, and amnion explants from three donors were infected with ZIKV (1.5 × 10^5^ PFU/mL) with or without prior incubation with human sera containing antibodies against DENV-1, DENV-2, or DENV-4. Four dpi ZIKV concentrations were quantified by real-time RT-PCR. Each column represents the median of 9 (ZIKV) or 27 explants (ZIKV + D) with interquartile range. Statistical analysis was performed with the Mann–Whitney test (*P *<* 0.05). **c**–**e** ZIKV infection kinetics in presence or absence of DENV-, YFV-, or CHIKV-immune sera, or naïve human serum. Placental villus (**c**), maternal decidua (**d**), and amnion (**e**) explants from four donors were infected with ZIKV (1.5 ×10^5^ PFU/mL) with or without prior incubation with human sera containing either antibodies against three different DENV serotypes, YFV or CHIKV, or a control serum. Virus concentrations of inocula 0 dpi and viral progeny 1, 2, 4, 6, and 8 dpi were quantified by real-time RT-PCR. All infections were done in triplicates for each placenta. Data points represent the mean of 12 explants per setting with SEM. Inocula were measured once per placenta and setting. *ZIKV* *+* *D1* ZIKV + DENV-1-immune serum, *ZIKV* *+* *D2* ZIKV + DENV-2-immune serum, *ZIKV* *+* *D4* ZIKV + DENV-4-immune serum, *ZIKV* *+* *YF* ZIKV + YFV-immune serum, *ZIKV* *+* *CH* ZIKV + CHIKV-immune serum, *ZIKV* *+* *S−* ZIKV + flavi- and alphavirus-naïve serum

To initially confirm that trophoblast cells are susceptible to ZIKV infection, we chose the placental cell lines HTR-8 and Swan71^21^ that are derived from primary cells isolated in the first trimester of pregnancies and morphologically represent trophoblast cells. Both cell lines amplified ZIKV RNA more than 10^5^-fold within 3 days post infection (dpi) at a multiplicity of infection (MOI) = 0.1 (Fig. 1a) and 4 dpi dead cells accumulated in the supernatant (Figure S1). VSC cells, isolated from villous stromal tissue and showing fibroblast morphology, amplified the virus 100-fold less efficiently (Fig. 1a). VSC were the only cell lines that did not exhibit visible cytopathic effect 5 dpi, whereas several choriocarcinoma cell lines with trophoblast-like morphology amplified ZIKV 10^4^–10^5^-fold (not shown) and exhibited severe cytopathic effects (Figure S1).

### Infection of placental explants—ADE of ZIKV replication

For a preliminary test of enhancement of ZIKV infection by DENV antibodies, fresh placental explant cultures from three placental and paraplacental compartments (placental villi, basal decidua, and amniochorionic membrane) were infected with ZIKV in the absence or presence of anti-DENV serotype 1-, 2-, or 4-immune sera. At 1 dpi, ZIKV replication was only seen in villous explants and only if these were infected in the presence of anti-DENV serum irrespective of DENV serotype. From 4 to 8 dpi, ZIKV replication was observed in explants representing all three compartments. The presence of any type of DENV-immune serum enhanced the speed of ZIKV replication, as well as the average virus concentrations in villous and decidual explants (Fig. 1b). Enhancing effects in amniochorionic explants were also visible, but not to an extent that was statistically significant.

To control against non-DENV-specific effects, we involved four additional placenta donors and included human sera containing antibodies against chikungunya virus (CHIKV) and YFV, as well as a serum from a flavi- and alphavirus-naïve patient (summarized in Fig. 1c–e). Plaque titrations were performed with the supernatants of selected replicates 4 dpi to show that differences in genome copy numbers corresponded to differences in the amount of infectious virus particles. Genome copy numbers were about 10^3^-fold higher than the number of viral plaque-forming units and genome copies consistently represented infectious virus titers (Figure S2).

**Fig. 2:**
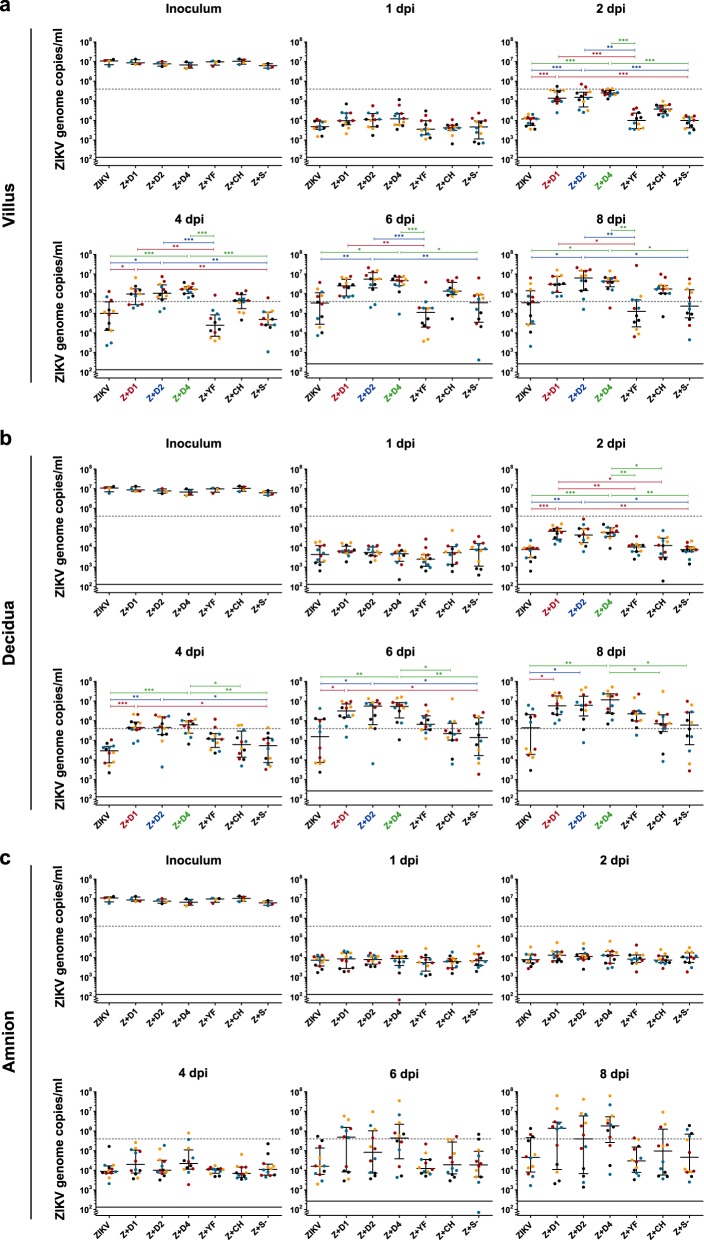
ZIKV replication in different placental tissue explants and ADE by DENV antibodies. Placental villus (**a**), maternal decidua (**b**), and amnion (**c**) explants were infected in triplicates with ZIKV (1.5 × 10^5^ PFU/mL) with or without prior incubation with human sera that either contained antibodies against one of three different DENV serotypes as indicated, YFV or CHIKV, or a control serum. The virus concentration of the inoculum 0 dpi and viral replication 1, 2, 4, 6, and 8 dpi were determined by quantitative real-time RT-PCR. Explants were obtained from four donors as indicated by black, blue, red, and yellow dots. Medians with interquartile ranges are presented for each treatment. The dashed line shows the threshold for successful infection used for the infection rate calculation in Table 1. The continuous line represents the detection limit of the real-time RT-PCR. Statistical analysis was performed with the Kruskal–Wallis test combined with Dunn’s multiple comparison test. Significant differences are indicated in red (Z + D1), blue (Z + D2), and green (Z + D4) (*P *<* 0.05, **P *<* 0.01, ***P *<* 0.001). *Z* *+* *D1* ZIKV + DENV-1-immune serum, *Z* *+* *D2* ZIKV + DENV-2-immune serum, *Z* *+* *D4* ZIKV + DENV-4-immune serum, *Z* *+* *YF* ZIKV + YFV-immune serum, *Z* *+* *CH* ZIKV + CHIKV-immune serum, *Z* *+* *S−* ZIKV + flavi- and alphavirus-naïve serum

In explants from all three placental compartments, average virus replication was enhanced in the presence of antibodies against any tested serotype of DENV. Enhancement was first observed in villous explants (day 1) and became apparent at 2 and 4 dpi in decidual and amniotic explants, respectively, confirming the results of the preliminary studies (Fig. 2). The differences in genome copy numbers between infections with ZIKV in the presence of DENV antibodies and ZIKV alone, or in the presence of a flavi- and alphavirus-naïve serum were significant from 2 dpi onward in the villous and decidual explants. There was variation between donors in their overall sensitivities to ZIKV infection, as well as in the extent of replication enhancement conferred by DENV-immune sera. These donor-specific variations were seen in all three tissue types (Fig. 2) and limited the number of tested immune sera, as all infections had to be performed in parallel per placenta.

Although ZIKV replication in the presence of CHIKV antibodies appeared to be faster in the villous explants compared with ZIKV alone, the CHIKV-immune serum did not enhance ZIKV replication significantly. The same applied for all other non-DENV-immune sera.

### ADE of ZIKV infection rates in placental explants

Because of donor-specific variabilities in susceptibility, we separately analysed the fractions of successfully infected explants per experimental setting (Table 1). In amniotic explants, the infection rate was 2.67-fold higher in the presence than in the absence of DENV antibodies. In decidual and villous explants, the infection rates were 1.42- and 1.81-fold higher, respectively, compared with infection without DENV antiserum. Differences were highly statistically significant for all three tissue types.

**Table 1:** ZIKV infection rates and ADE in different placental tissue explants

	Successfully infected explants										Relative risk (Fisher’s exact test)	
	ZIKV	Z + S −	Z + CH	Z + YF	Z + D1	Z + D2	Z + D4	Z + C^1^	Z + nD^2^	Z + D^3^	Z + D/ Z + C	Z + D/ Z + nD
Amnion	3/12 (25%)	4/12 (33%)	3/12 (25%)	1/12 (8%)	7/12 (58%)	6/12 (50%)	9/12 (75%)	7/24 (29%)	11/48 (23%)	22/36 (61%)	2.1 (0.0193)	2.67 (0.0006)
Decidua	6/12 (50%)	6/12 (50%)	9/12 (75%)	11/12 (92%)	12/12 (100%)	10/12 (83%)	12/12 (100%)	12/24 (50%)	32/48 (67%)	34/36 (94%)	1.89 (0.0001)	1.42 (0.0025)
Villus	5/12 (42%)	5/12 (42%)	11/12 (92%)	4/12 (33%)	12/12 (100%)	11/12 (92%)	11/12 (92%)	10/24 (42%)	25/48 (52%)	34/36 (94%)	2.27 ( < 0.0001)	1.81 ( < 0.0001)

^1^Sum of ZIKV and Z + S −; ^2^Sum of all settings without DENV serum; ^3^Sum of all settings containing DENV sera. *Z* *+* *D1* ZIKV + DENV-1-immune serum, *Z* *+* *D2* ZIKV + DENV-2-immune serum, *Z* *+* *D4* ZIKV + DENV-4-immune serum, *Z* *+* *YF* ZIKV + YFV-immune serum, *Z* *+* *CH* ZIKV + CHIKV-immune serum, *Z* *+* *S* *−* ZIKV + flavi- and alphavirus-naive serum.

## Discussion

Here we present observational evidence of an enhancement of placental ZIKV infection in vitro in the presence of DENV antibodies. In previous studies on ZIKV placental explants, only ZIKV without the presence of DENV antibodies was investigated^19,22,23^. In the present study we find an earlier start of placental ZIKV replication and higher infection rates in the presence of DENV-immune sera. The earlier start of replication is consistent with the hypothesis of an increased uptake of complexes of non-neutralizing antibodies and virions in Fc-receptor-bearing cells due to ADE. Due to the ethical and logistical challenges in obtaining placental material, we have not been able to confirm the exact mechanism of the observed ADE phenomena, in particular the involvement of Fc receptors. However, previous work has shown that the villous stroma supports ZIKV replication, while the syncytiotrophoblast is resistant to ZIKV infection^19^. Fcγ-mediated ADE seems possible because the stroma contains cytotrophoblasts, fetal macrophages (Hofbauer cells), fibroblasts, and endothelial cells that all express Fcγ receptors^19,24^. Infection in first term placental explants is known to target proliferating cell column cytotrophoblasts and Hofbauer cells^20^. To reach these susceptible cells, ZIKV virions bound to cross-reactive antibodies may cross the syncytiotrophoblast layer in a similar way as human cytomegalovirus, based on virion-IgG complexes that are transported across the syncytiotrophoblast layer by FcRn-mediated transcytosis^25^. We suspect that our observed ADE phenomena also follows an Fc-receptor-dependent mechanism, but have to caution that this will require further study.

Our observation that ZIKV ADE was strongest in chorionic villous explants corresponds to the high density of Fc-receptor-bearing cells in that compartment. Decidual explants also showed clear ADE, and it is known that decidual macrophages express Fc receptors and are susceptible to ZIKV infection^22,26^. Decidual explants contain the extravillous trophoblasts in the anchoring villi that invade the uterine wall and are in direct contact with maternal decidual and immune cells^23^. It is plausible for these cells to be involved in Fc-receptor-mediated ADE, even though the decidual explants also include partly villous tissue, so that an enhancement effect could also be due to infection of these cells.

Paraplacental transmission through the amniochorionic membranes is another way for ZIKV to reach the fetus. This potential route of transmission is supported by the detection of ZIKV in amniotic fluid^27^. Little is known about the expression of Fc receptors at the amniochorionic membrane itself, but cells in the decidual layer associated with the amniochorionic membrane express Fcγ receptors and therefore could support ADE^28^. Based on our results, infection and ADE in the paraplacental compartment seems likely, but less likely than in the placental compartment.

Our findings show that DENV-specific ADE occurs during ZIKV infection of the placenta in vitro. The specificity of the effect for DENV-immune sera corresponds to the phylogenetic relatedness between DENV and ZIKV E proteins^11^ and implies that the enhancement is not derived from unspecific serum components. Although not of the same titer, YFV antibodies did not enhance ZIKV infection either. A limitation of our study was the number of human sera we could test. Experiments had to be conducted in parallel per placenta to account for variations between placenta donors. The impact of antibody titers or types and number of previous infections with DENV requires further studies.

At present, sufficiently powered clinical studies addressing the enhancement of ZIKV fetal infection by previous dengue infection are not available. In the only available study to date, no such association was identified but the inclusion of a high proportion of DENV-seropositive mothers (87.5%) limited the statistical power with regard to DENV-dependent ADE^29^. Moreover, only mothers with symptomatic ZIKV infection were included, which may have selected for severe courses with higher rates of fetal infection. Also in experimental studies in primates, ADE of fetal infection has been difficult to assess, because vertical transmission in macaques seems to be considerably more effective than in humans. The only study addressing DENV-associated fetal damage by ZIKV was a study on five pregnant macaques infected with ZIKV, of which three animals received monoclonal antibodies against DENV. Neuronal malformations were observed in all fetuses including those without administration of DENV antibodies, preventing conclusions on ADE^30^.

Although the present study is limited to in vitro infection, the results provide a reminder of the possibility that mothers previously infected with DENV may be at an increased risk of transmitting ZIKV to their fetus. Increased placental infection rates may correlate with an increase of fetal malformations.

## Materials and methods

### Virus strain and cells

The ZIKV strain H/PF/2013 from French Polynesia, which belongs to the recent epidemic lineage, was used in this study. The virus was grown in C6/36 cells and titrated by plaque assay on Vero cells (titer: 1.5 × 10^7^ PFU/mL). Three human placental cell lines were used for virus growth kinetics. HTR-8/SVneo (ATCC® CRL-3271™) and Swan71^21^ are immortalized first trimester trophoblast cell lines and VSC is a primary villous stromal cell line. HTR-8/SVneo and Swan71 cells were obtained from the Department of Obstetrics, University of Jena. VSC cells were obtained from the Department of Developmental Pathology, University of Bonn. VSC cells were maintained in Dulbecco’s modified Eagle’s medium (DMEM) (Gibco®, Thermo Fisher Scientific, Waltham, MA, USA) containing 10% fetal calf serum (FCS) (Biochrom, Berlin, Deutschland) and 2 mM glutamine (Gibco®). Swan71 cells were cultivated in DMEM medium containing 10% FCS. HTR-8/SVneo cells were cultivated in RPMI medium (Gibco®) containing 10% FCS. All cells were incubated at 37 °C with 5% CO_2_.

### Cell culture growth kinetics

Cells were seeded in 24-well plates 1 day before infection (6 × 10^4^ cells per well for VSC and 3 × 10^4^ cells per well for all other cell lines). The cells were infected with ZIKV in triplicates at MOI 0.1 in 300 µl DMEM without additives and incubated for 1 h at 37 °C. After incubation the cells were washed two times with 1 mL phosphate-buffered saline (PBS) (Gibco®) and once with medium with additives. Finally, 1 mL medium with additives was added and 75 µl supernatant was transferred into 300 µl RAV1 buffer for RNA extraction (0 dpi). Further samples were taken every 24 h for 3 dpi. The cells were observed daily until 5 dpi for signs of cytopathic effects.

### Human sera

The DENV-1-, -2-, and -4-immune sera were provided by the Bernhard Nocht Institute for Tropical Medicine in Hamburg. All sera stemmed from German travellers with primary DENV infection. IgG titers in immunofluorescence were 81,920 for the DENV-1-positive serum and 20,480 for both the DENV-2- and DENV-4-positive sera. A DENV-3-immune serum was not included, because we did not have access to sufficient volume of serum at the time of this study. The CHIKV- and YFV-immune sera, and a flavivirus- and alphavirus-naïve serum were defined by immunofluorescence and enzyme-linked immunosorbent assay tests, and were provided by the Institute of Virology in Bonn. The CHIKV-immune serum had a titer > 100 and the titer of the YFV-immune serum was > 200. The YFV-immune serum stemmed from a donor who was vaccinated against YFV.

### Ethics statement

The study was approved by the ethics board of the Medical Faculty, University of Bonn (ethics vote number 252/16). All women included in this study provided written informed consent.

### Preparation and infection of placental tissue explants

To obtain tissue explants from amniochorionic membrane, placental villi, and maternal decidua, placentae from healthy mothers with term delivery by cesarean section were used. All tissue explants were washed in PBS with 1% penicillin–streptomycin and 1% gentamycin (Biochrom), and then cut into smaller pieces (3–4 mm in diameter). Each explant was placed in a separate well of a 24-well plate. Before infection, RPMI medium containing 10% FCS, 1% penicillin–streptomycin, 1% gentamycin, 1.5 × 10^5^ PFU/mL of ZIKV, and human serum diluted 1:1000 was incubated at 37 °C for 1 h. Each explant was then infected with 1 mL of the prepared inoculum and incubated for 24 h at 37 °C with 5% CO_2_. 75 µl of the inoculum was transferred into 300 µl of RAV1 buffer to determine the virus concentration in the inoculum. All infections were done in triplicates for each placenta. At 1 dpi, the explants were washed three times with PBS containing antibiotics. 2 mL of RPMI medium containing 10% FCS, 1% penicillin–streptomycin, and 1% gentamycin was added, and 75 µl of the supernatant was transferred into 300 µl of RAV1 buffer. Samples were taken at 1, 2, 4, 6, and 8 dpi. After taking the 4 dpi sample, half of the medium was replaced with fresh medium.

### Quantification of ZIKV genome copies

RNA extraction was performed using the NucleoSpin® RNA Virus kit from Macherey-Nagel (Düren, Germany) according to the manufacturer’s instructions, except that initial incubation at 70 °C was extended to 10 min and RNA was eluted in 50 µl of H_2_O. The concentration of ZIKV genomes was measured by real-time RT-PCR targeting the E gene^31^.

### Statistical analysis

To calculate the infection rate of the placental explants, minimal ZIKV genome concentrations were applied as criteria for successful infection. The threshold was set to 4 × 10^5^ genome copies/mL at 8 dpi, because this was higher than the concentration measured in any of the explants at 1 dpi and more than 1 log higher than the mean at 1 dpi in a preliminary experiment. Virus loads were also tested on days between 1 and 8 dpi. To analyse the effect of antibodies on infection rates the Fisher’s exact test was used. In addition, the Mann–Whitney test and the Kruskal–Wallis test with Dunn’s multiple comparison for all pairs as post-hoc test were used to compare ZIKV replication in settings with and without DENV-immune sera. The statistical analyses were performed in GraphPad Prism 7.

## Electronic supplementary material

Figure S1

Figure S2

Supplementary Figure Legends
