# Supplementary figures and images for: Zika virus infection in human placental tissue explants is enhanced in the presence of dengue virus antibodies in-vitro
Source: Emerg Microbes Infect. 2018 Dec 1;7:198. doi: 10.1038/s41426-018-0199-6 (PMC6274641; doi:10.1038/s41426-018-0199-6)

**Mock**

**MOI 0,1**

**MOI 1**

**HTR-8**

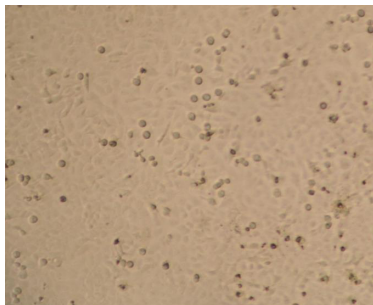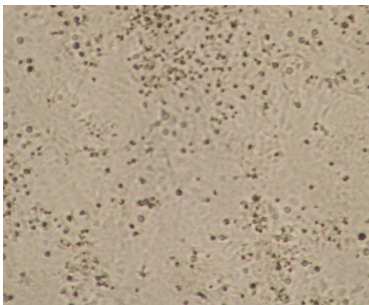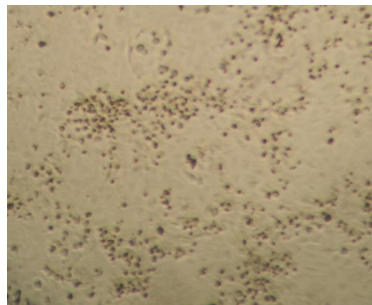

**Swan 71**

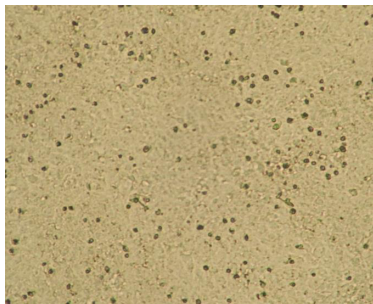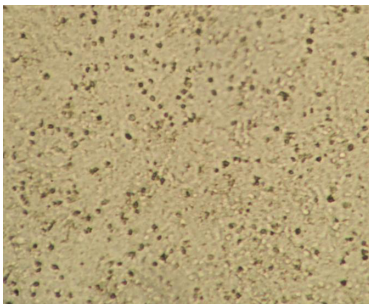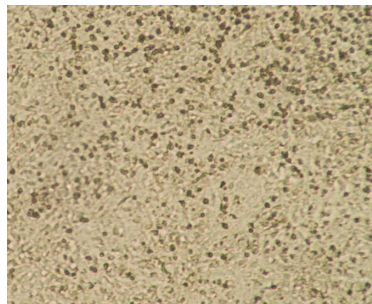

**BeWo**

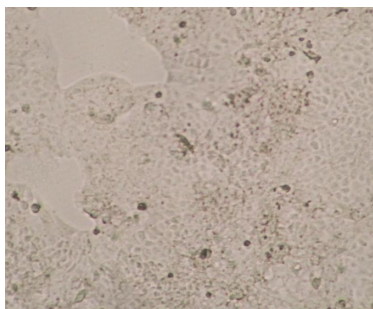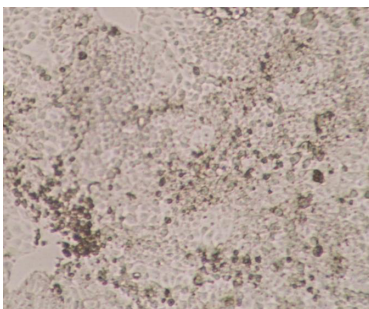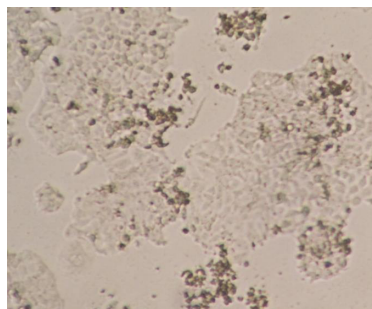

**JEG-3**

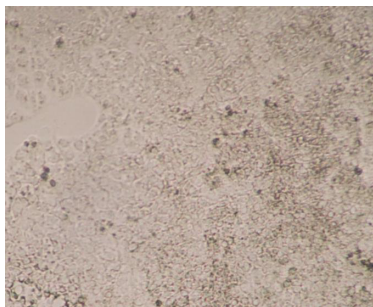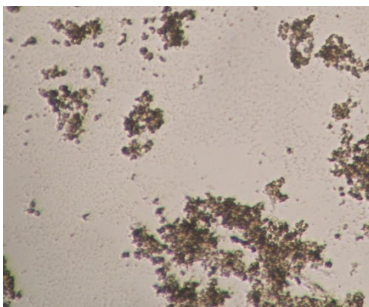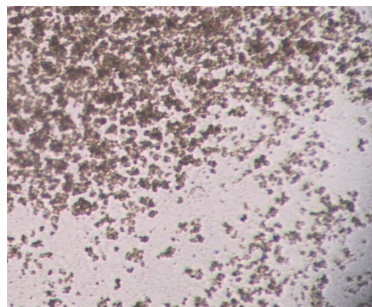

Supplement: Supplementary file 1 — Figure S1 [file 41426_2018_199_MOESM1_ESM.pdf]

4 dpi

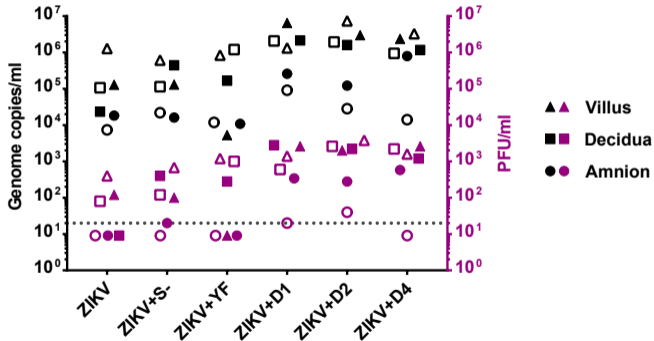

Supplement: Supplementary file 2 — Figure S2 [file 41426_2018_199_MOESM2_ESM.pdf]
