# Supplementary material for: Zika virus infection in human placental tissue explants is enhanced in the presence of dengue virus antibodies in-vitro
Source: Emerg Microbes Infect. 2018 Dec 1;7:198. doi: 10.1038/s41426-018-0199-6 (PMC6274641; doi:10.1038/s41426-018-0199-6)
Supplement: Supplementary file 3 — Supplementary Figure Legends [file 41426_2018_199_MOESM3_ESM.docx]

**Figure S1: Cytopathic effects in placental cell lines infected with ZIKV.** Immortalised trophoblast cell lines (HTR-8 and Swan 71) and choriocarcinoma cell lines (BeWo and JEG-3) were either mock infected or infected with ZIKV at MOI 0.1 or 1. Photographs were taken 4 dpi to document cytopathic effects.

**Figure S2: Virus production in placental tissue explants.** Production of infectious virus progeny was determined 4 dpi by plaque titration on Vero cells. For two placenta donors (indicated by filled and open symbols, respectively) one experimental replicate each, containing either ZIKV without serum, or ZIKV with human serum anti DENV-1, -2, or -4, YFV or a control serum, respectively, were tested. Genome copy numbers and PFUs are shown in black and purple, respectively. The dashed line indicates the detection limit of the plaque assay. Abbreviations: ZIKV+D1 – ZIKV + DENV-1-immune serum, ZIKV+D2 – ZIKV + DENV-2-immune serum, ZIKV+D4 – ZIKV + DENV-4-immune serum, ZIKV+YF – ZIKV + YFV-immune serum, ZIKV+S- – ZIKV + flavi- and alphavirus-naïve serum.
